# Robotic-Assisted Surgery for Benign Urological Conditions

**DOI:** 10.1100/tsw.2006.398

**Published:** 2006-06-20

**Authors:** Declan G. Murphy, Ben J. Challacombe, Lail-U-Mah Zaheer, M. Shamim Khan, Prokar Dasgupta

**Affiliations:** Department of Urology, Guy's Hospital & GKT Medical School, London, UK

**Keywords:** robotic, urology, pyeloplasty, reconstruction

## Abstract

Robotic technology for use in surgery has advanced considerably in the past 10 years. This has become particularly apparent in urology where robotic-assisted radical prostatectomy using the da Vinci surgical system (Intuitive Surgical, CA) has become very popular. The use of robotic assistance for benign urological procedures is less well documented. This article considers the current robotic technology and reviews the situation with regard to robotic surgery for benign urological conditions.

## INTRODUCTION

Urologists have been very quick to embrace some of the most exciting technological advances in surgery over the past 10 years. Though as a specialty we were somewhat reticent about joining the laparoscopic revolution in the 1980s, it is clear that some of the most innovative advances in minimally invasive surgery in recent years have been spearheaded by the urologists. Nowhere is this better demonstrated than the proliferation in robot-assisted surgery, especially robot-assisted laparoscopic radical prostatectomy. In this review, we will recap some of the advances in robotic-assisted surgery, but will focus mainly on the current state of robotic-assisted surgery for benign rather than malignant urological conditions.

### Definitions

A surgical robot has been defined as "a computer-controlled manipulator with artificial sensing that can be reprogrammed to move and position tools to carry out a range of surgical tasks"[1]. Strictly speaking, the current popular surgical "robots" do not satisfy this definition. Hence, some authors have suggested the term "computer-assisted surgery" to describe more accurately the current generation of robotic devices[2]. Whatever the conclusion of that pedantic debate, the term "robotic" is in popular use to describe the range of technology under discussion here.

## ROBOTIC TECHNOLOGY IN UROLOGY

The early pioneers in this field included Wickham et al. from Guy's Hospital and Imperial College, London, who developed the PROBOT in the late 1980s. The PROBOT used a robotic frame that guided a rotating blade to complete transurethral resection of the prostate (TURP). Initial studies on prostate-shaped potatoes were followed up by clinical trials in patients to demonstrate safety and feasibility of the technology[3]. This was a truly autonomous device, satisfying the definitions outlined above. However, convincing differences over conventional TURP were not demonstrated.

Subsequently, the percutaneous renal access robot, PAKY-RCM, demonstrated superior accuracy, but longer operating (access) times when compared to humans in a randomised control trial of transAtlantic telerobotics[4]. This remains the only randomised control trial of robotic vs. nonrobotic technology for surgical applications. Kavoussi's group also published on their experience with remote "telerounding" robots[5]. However, though interesting and thought provoking, the technologies described are yet to be established in routine clinical practice.

### Robotic Laparoscope Manipulators

The development of laparoscope manipulators such as the Automated Endoscopic System for Optimum Positioning (AESOP™) and EndoAssist™ has certainly found a niche in laparoscopic urological procedures. These devices hold the laparoscope under voice, pedal, or infrared motion control and provide steadier images with less instrument collisions than a human assistant[6]. These are particularly useful in procedures such as laparoscopic radical prostatectomy, freeing the assistant to use two ports, while ensuring very steady images. They also enable the concept of solo surgery, dispensing with the need for surgical assistants[7].

### Master-Slave Systems

It is the da Vinci™ surgical system (Intuitive Surgical, CA) that has generated the most headlines with regard to robotic-assisted surgery (Fig. 1). It was developed in the late 1990s, while a competitor, the ZEUS™ system, was also in existence. This is a master-slave system rather than a true autonomous robot. The surgeon sits at a console remote from the patient, controlling three or four robotic arms, which are docked through the laparoscopic ports. Three-dimensional (3D) vision, 7 degrees of freedom (DoF) of movement, and intuitive movements of the robotic instruments are among its proposed benefits over conventional laparoscopic surgery. The da Vinci™ technology, its advantages, and disadvantages are described in detail elsewhere[8].

The tremendous increase in the number of robotic procedures reported worldwide is largely due to the increasing acceptance of robotic-assisted radical prostatectomy (RALP) for the treatment of localised prostate cancer. From about 1500 robotic procedures in 2000, over 20,000 procedures were performed in 2004[9]. Urology accounts for the largest single-speciality increase, with over 8000 robotic prostatectomies performed in 2004. RALP now accounts for over 10% of radical prostatectomies performed in the U.S., a proportion that is increasing year on year. We shall now review the increasing role of robotic-assisted surgery for benign urological conditions.

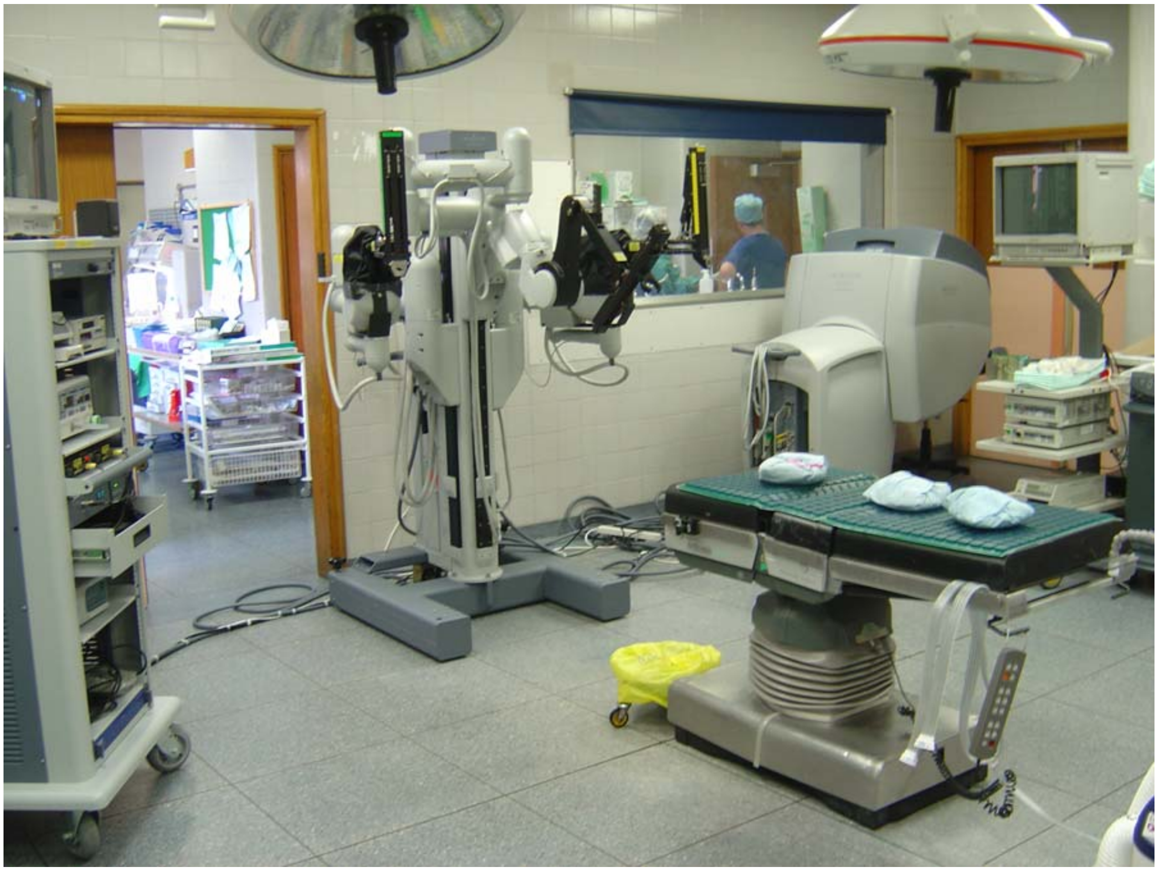

**FIGURE 1**. da Vinci™ surgical system at Guy's Hospital.

## ROBOTIC-ASSISTED SURGERY FOR BENIGN UROLOGICAL CONDITIONS

### Why Use the da Vinci™?

There are putative reasons why the technology inherent in this system should have specific benefits for certain procedures. Broadly speaking, the combination of 3D vision and extra DoF should decrease the difficulty associated with laparoscopic suturing, thereby encouraging the use of the da Vinci™ for reconstructive procedures. This is already apparent with the reduced learning curve reported for RALP[10].

By extension, one could apply the same rationale to procedures such as dismembered pyeloplasty, ureteric reimplantation, and other reconstructive procedures. However, for ablative procedures such as nephrectomy, it is less clear where the advantage of using this technology may lie.

## PROCEDURES REPORTED TO DATE

### Pyeloplasty

The benefits of minimally invasive surgery are immediately apparent for patients undergoing laparoscopic dismembered pyeloplasty. However, this remains a challenging procedure with prolonged operating times, even in the larger series[11]. Robotic-assisted pyeloplasty has been reported in adult and paediatric populations, the latter using 5-mm robotic ports. Success rates equal that of laparoscopic and open pyeloplasty[12]. The largest reported series (n = 50) describes operative times of 122 min with anastomotic times averaging only 20 min[13].

Regarding the learning curve, Bernie et al. examined the first seven robotic and first seven standard laparoscopic pyeloplasties performed by a single surgeon at his unit and concluded that there was no significant difference[14]. Operating times averaged 5.4 h for the robotic and 5.2 h for the standard laparoscopic group. Clearly, a larger, prospective, randomised trial would be necessary to support this conclusion. Bentas et al. completed 11 laparoscopic pyeloplasties using the da Vinci™ system, having had no previous laparoscopic experience[15]. Operating time averaged 197 min and 100% success was reported at 12-month follow-up.

However, little high-quality evidence exists to prove whether this technology offers better outcome compared to standard treatments (laparoscopic, endoscopic, or open). Indeed, conflicting conclusions are drawn from two studies that have sought to compare robotic-assisted pyeloplasty with standard laparoscopic pyeloplasty. In a nonrandomised comparison, Gettman's group reported shorter operative and anastomotic times compared to pure laparoscopic pyeloplasty[16]. The total operative and suturing times were 140 and 70 min compared to 235 and 120 min for robotic and laparoscopic pyeloplasty, respectively. However, Kavoussi's group has reported longer operating times and significantly higher costs associated with the robotic approach in a small comparative trial (n = 20)[17]. Operative costs were 2.7 times higher in the robotic group (1.7 times if the capital costs were excluded).

## Nephrectomy: Simple/Radical/Donor/Partial

A number of early reports described the use of robotic manipulators such as AESOP™ to facilitate laparoscopic nephrectomy[18,19]. Gill et al. described their experience using the Zeus™ master-slave system to perform laparoscopic nephrectomy on five pigs[20]. However, it was Guilloneau et al. who reported the first robotic-assisted laparoscopic nephrectomy on a human in 2001, again using the Zeus™ system[21].

Since then, a considerable number of reports of robotic-assisted ablative renal surgery have been published, none of which have shown any significant advantage over conventional laparoscopic techniques. As with all new equipment, early reports focussed on ascertaining the safety aspect of robotic technology in performing the existing procedures. Hence, reports of robotic-assisted nephroureterectomy[22], radical nephrectomy[23], and donor nephrectomy[24] soon followed Guilloneau's initial report of simple nephrectomy. However no large prospective trials, or even large nonrandomised series, have followed these initial reports, prompting commentators to suggest that there is "an absence of perceived benefit with purely ablative procedures"[25], especially when one considers the widespread acceptance of laparoscopic techniques in such situations. Despite this, there are ongoing reports of robotic-assisted donor nephrectomy, some of which may be encouraged by the prospect of a reduced learning curve for surgeons with no previous laparoscopic experience[26]. A prospective, randomised trial would help to answer these questions.

One could argue that the role of robotic assistance for laparoscopic partial nephrectomy may be somewhat different due to the significant reconstructive element to these procedures. Phillips et al. reported their experience of 12 robotic-assisted partial nephrectomies, using the da Vinci™ system, concluding that the procedure was "safe, feasible, and reproducible", despite converting 2 procedures (1 to open; 1 to hand-assisted laparoscopy) due to bleeding[27]. A third procedure was converted to the standard laparoscopy due to robot malfunction. Warm ischaemia time was 26 min. Gettmann et al. reported 13 robotic-assisted partial nephrectomies with no conversions and an average blood loss of 170 ml[28]. Eight patients in this series underwent intra-arterial cooling (ischaemia time 33 min) while five underwent hilar clamping without cooling (ischaemia time 22 min). These data are not conclusive in favour of robotic assistance for partial nephrectomy at this stage.

## Adrenalectomy

The first robotic-assisted adrenalectomy in a human was reported in 2001[29], followed by sporadic reports of small series[30,31,32,33]. Brunaud et al. compared 14 robotic-assisted laparoscopic adrenalectomies with 14 using the standard laparoscopic approach[34]. Operative times remained longer in the robotic group with no advantage demonstrated over the standard laparoscopic approach.

## Ureteric Reimplantation

The reconstructive element to surgery for vesicoureteric reflux has attracted a little attention from the paediatric urologists. Peters has described extravesical, Lich-Gregoir, ureteric reimplant using the da Vinci™ system in 19 patients with a satisfactory outcome[35]. He subsequently elegantly described robotic-assisted, Cohen cross-trigonal, ureteric reimplantation via the transvesical approach[36]. The robot was docked into the bladder and maintained in position using $CO_2$-induced pneumovesicum. Six patients underwent the procedure with one postoperative urine leak through inadequate port-site closure. Low-grade reflux persisted in one patient who did not require further intervention.

## Ileal Conduit and Cystoplasty

Again the reconstructive element of this procedure has attracted some attention from institutions with the da Vinci™ surgical system. Ileal conduit with totally intracorporeal, ureteroileal anastomosis took 10 h in one case report[37]. The same group reported similar operating times for two further patients undergoing the same procedure for radiation-induced cystitis[38]. When cystectomy was also done in their series, the operating time reached 13.8 h. Hubert et al. reported robotic-assisted, laparoscopic, intracorporeal cystectomy and ileal conduit formation in two men with neurogenic bladder[39]. Operative times were 9.25 and 6.75 h, respectively. Orthotopic bladder reconstruction has also been reported with an operating time of 8.5 h[40].

The most striking aspect of all these reports is the very long operating times associated with these procedures. These are in keeping with the early reports of Gill et al. of laparoscopic cystectomy and ileal conduit with totally intracorporeal anastomosis[41] when operating times averaged 11 h. It is noteworthy that Gill and others now favour laparoscopic cystectomy with urinary diversion performed extracorporeally to reduce operating times (personal communication). It is the authors' preference to perform robotic-assisted, radical cystectomy with ileal conduit or neobladder formation performed extracorporeally[42].

## Miscellaneous Others

Khan et al. from London are reporting the first experience of robotic-assisted colposuspension shortly (in press, *J. Endourol.*). The da Vinci™ system has been used for sacrocolpopexy in 31 patients[43]. Fleming has described robotic-assisted vasovasostomy in a case report[44]. Hoznek et al. have docked the da Vinci™ into the open wound of a renal transplant recipient to facilitate the vascular and ureteric anastomoses[45]. Kaouk et al. have reported the technical feasibility of robotic-assisted sural nerve grafting using the da Vinci™ system with a variable outcome[46]. These procedures are unlikely to attract widespread acceptance at the present time.

Table 1 summarises the more common robotic-assisted procedures for benign urological conditions reported to date.

**TABLE 1**
**Robotic-Assisted Surgery for Benign Urological Conditions**

| Procedure | Ref. | Indication | Number | Operating Time (min) | Remarks |
|---|---|---|---|---|---|
| Pyeloplasty | Patel[13] | UPJ obstruction | 50 | 122 | 100% success at 11 months |
| | Gettmann et al.[16] | UPJ obstruction | 6 | 140 | No complications |
| Nephrectomy | Hubert et al.[23] | Simple, radical, and donor | 16 | 110 | One conversion to open surgery |
| | Renoult et al.[26] | Donor | 13 | 185 | One DVT |
| | Gettmann et al.[28] | Partial nephrectomy | 13 | 215 | Average blood loss 170 ml |
| Adrenalectomy | Brunaud et al.[34] | Benign mass | 14 | 111 | One conversion to open surgery |
| Ileal conduit | Hubert et al.[39] | Neurogenic bladder | 2 | 480 | Includes time for cystectomy |

## CONCLUSIONS

The published literature regarding robotic-assisted urological procedures is dominated by references to robotic-assisted laparoscopic radical prostatectomy, a procedure gaining increasing acceptance. The literature pertaining to robotic-assisted surgery for benign urological conditions is dominated by case reports and small cohort series, making it difficult to draw conclusions about the merit or otherwise of robotic procedures described thus far. However, it seems likely that robotic assistance may have at least a useful role in laparoscopic pyeloplasty. Whether it shortens the learning curve, especially for laparoscopic novices, remains to be seen. It is certainly our experience that robotic-assisted pyeloplasty reduces anastomotic times and allows shorter postoperative hospital stay[47].

The role of robotic assistance for other procedures remains to be seen. It appears unlikely that ileal conduit diversion and neobladder formation will attract widespread appeal while operating times remain very long. Laparoscopic partial nephrectomy is a procedure performed by those with advanced laparoscopic skills who therefore are less likely to benefit from robotic assistance. A number of groups are performing robotic-assisted donor nephrectomy and it would be very helpful indeed if a randomised trial comparing robotic-assisted with the conventional laparoscopic approach were to emerge from one of these centres.

Of course, the role of robotic assistance for all surgical procedures remains in its infancy. This article has reviewed the relevant current literature, largely pertaining to the da Vinci™ system, which remains among the first generation of master-slave robotic devices. We should look forward to future generations of robotic devices thath may open up many other exciting applications within the surgical specialities.

## ACKNOWLEDGMENTS

We wish to thank the Charitable Foundation of Guy’s & St Thomas’ Hospital.

## BIOSKETCH

**Declan Murphy** is a Specialist Registrar in Urology at Guy's Hospital in London, and is the Fellow in Laparoscopic and Robotic Urology.
